# Implementing point-of-care ultrasound (PoCUS) in UK primary care: current and future perspectives

**DOI:** 10.3399/BJGP.2026.0221

**Published:** 2026-06-25

**Authors:** Aaron Poppleton, Lee Pursglove, Ahmed Mostafa, Ibrahim ElNaggar, Jay Jadeja, Pascual Daza-Ramirez

**Affiliations:** 1 School of Medicine and Dentistry, University of Lancashire, Preston, UK; 2 School of Medicine, Keele University, Keele, UK; 3 GP2Health, Private GP Clinic, Morpeth, UK; 4 Shropshire Community MSK Service, Telford, UK; 5 Virtual Care GP, Royal Berkshire Hospital/OOH GP, Reading, UK; 6 Royal Free Hospital, London, UK; 7 VTS, Health Education England, Cornwall, UK

Medical history taking and physical examination are increasingly being complemented by advanced diagnostics including imaging in community healthcare settings. Point-of-care ultrasound (PoCUS) is a portable, non-invasive, real-time test performed by the treating clinician to answer focused diagnostic questions or guide common procedures. Integration of PoCUS within the primary care consultation can enhance diagnostic assessment and interventions, improving timeliness of care.^1^ Despite widespread use in UK hospital services and primary care systems in Europe, America, and Australia, PoCUS is underutilised in UK primary care, where its value, safety, and cost-effective implementation are yet to be realised.^2^ As the Royal College of General Practitioners (RCGP) Ultrasound Special Interest Group (SIG), we present how PoCUS could influence UK primary care in the short- to medium-term.

## Development of PoCUS

Ultrasound has supported health care since the 1950s. PoCUS, when used appropriately, can reduce primary care referral rates for imaging, specialist opinion, or admission to hospital, with potential for reductions in healthcare costs and carbon footprint.^3^ In visualising pathology, PoCUS can improve accuracy of diagnosis, reducing need for repeated consultation and treatment,^2^ while enhancing patients’ understanding, trust, and involvement within the care process.^4^ Device portability raises the potential to facilitate community-based care for populations experiencing geographical, social, or financial barriers to accessing secondary care, including rural, housebound, homeless, and socioeconomically disadvantaged or vulnerable individuals.

The NHS 10-Year Health Plan emphasises moving care from hospitals to the community, digital transformation of services, and improving disease prevention. These aspirations align closely with PoCUS-enabled care, but would require questions around widespread adoption within UK primary care to be addressed,^5^ including:

- limited structured PoCUS training, mentorship, and supervision by experienced practitioners;
- unclear and complex local policies for accreditation, documentation, image storage, and quality assurance;
- limited access to ultrasound equipment and funding;
- heavy clinician workload limiting protected time for upskilling, practice, and maintenance of competence; and
- variable acceptance among practising GPs and other medical specialties.

We present the current situation and proposed steps to overcome these barriers to implementation, training, research, and governance of PoCUS within UK primary care.

## PoCUS in UK primary care

PoCUS is endorsed by the World Organization of Family Doctors (WONCA) for all GPs in the European Region.^6^ A core curriculum lists 40 high-value, clinically relevant, targeted examinations requiring ≤5 minutes (for example, gallstones, hydronephrosis, Baker’s cyst, intrauterine contraceptive device placement, deep vein thrombosis), alongside training standards, competencies, supervision, and quality assurance.^7^

Training pathways vary, including university-based Consortium for the Accreditation of Sonographic Education (CASE) programmes, in-person and remote primary-care-focused short courses, and preliminary locality level GP training (Table 1). The RCGP Ultrasound SIG (established 2024) has brought together local initiatives and networks to identify and promote best practice in PoCUS training and competency in the UK. Its virtual community of practitioners (>200 members) provides a forum for news, questions, case discussions, and resource development, including RCGP fringe events, conference workshops, podcasts, and publications for current and future PoCUS users. While limited GP time/motivation, trainer numbers, and an already expansive GP curriculum are obstacles to adoption, PoCUS training has been documented to improve job satisfaction, re-energise/prevent burnout, and increase attractiveness of general practice for some primary care clinicians.^8^ We envisage that GPs would maintain ultrasound competency through periodic training updates, mentoring, locality peer-CPD groups, auditing scan results and outcomes, and reflective practice. Regional GP PoCUS experts could support and train practice-level PoCUS champions to support colleagues’ use and skill development. We recognise the time, training, and equipment requirements for PoCUS will mean that uptake may not be practical for all GPs within NHS primary care currently.

**Table 1.** Generalist and dedicated GP-oriented courses

Course	Description	Website	Accreditation
Orca Medical	A blended remote and face-to-face GP-focused PoCUS course delivered in two phases with mentorship and a loaned ultrasound device	https://orcamedical.co.uk/product/point-of-care-ultrasound-for-community-practise-part-1/	RCP (UK)
Primary Care PoCUS (PCP) Course	Consisting of 2 face-to-face days of learning and 9 months of remote cloud-based mentorship	https://primarycarepocus.co.uk/	Not accredited
Bromley Primary Care One Course	Introduction to ultrasound applications in primary care. Includes simulators and ultrasound devices. Optional PgCert	https://www.bromleyemergency.com/our-courses/ultrasound-for-primary-care/	PgCert
Global Ultrasound Institute (GUSI) UK	Longitudinal 12-month fellowship blending in-person with online learning. Personal scan review mentorship. Comprehensive curriculum	https://globalultrasoundinstitute.com/en/gusi-in-the-united-kingdom/	CME, AAFP, and EACCME
Universities (CASE-accredited programmes)	Formal university-based ultrasound training over an academic year, providing gold-standard CASE-accredited training	https://www.case-uk.org/course-directory/	CASE
Yeovil Locality VTS programme, Somerset	Locality level GP training programme between the radiology department at Yeovil District Hospital and the GP practices in Symphony Healthcare Services. Uses Royal College of Radiologists’ training guidelines, scanning logbooks, audit, and reflective practice	doi: 10.3399/bjgp21 × 714677	Royal College of Radiologists’ training guidelines
Other PoCUS Accreditation Pathways	Lists established specialty-based PoCUS training with overlap with primary care, such as RCEM, BSE, SAM, and the ICS courses, for example, FAMUS and FUSIC	https://www.pocusuk.org/accreditation-pathways	Faculty specific
Self-directed learning	Self-directed learning using personal study and practice without formal structured training	—	Not accredited

AAFP = American Academy of Family Physicians; BSE = British Society of Echocardiography; CASE = Consortium for the Accreditation of Sonographic Education; CME = continuing medical education; EACCME = European Accreditation Council for Continuing Medical Education; FAMUS = Focused Acute Medicine Ultrasound; FUSIC = Focused Ultrasound in Intensive Care; ICS = Intensive Care Society; RCEM = Royal College of Emergency Medicine; RCP = Royal College of Physicians; SAM = Society for Acute Medicine;

### Clinical practice

Members of the RCGP Ultrasound SIG use PoCUS for varied applications within primary care, shaped by clinician interest, training, capacity, and patient need. Applications beyond the core GP PoCUS curriculum include:

- **Complementing special interests**. PoCUS is being used in GP musculoskeletal clinics to visualise, differentiate, and categorise degree of shoulder pathologies and identify conditions requiring rapid onward referral, such as inflammatory arthritis. Ultrasound guidance is facilitating intra-articular steroid injections, hydrodilatation, and assessment of treatment effect.
- **Hospital step-down/virtual care** of chronic disease management and admission avoidance. Examples include fluid status/diuretic titration in heart failure, monitoring of intra-abdominal collections in cancer, and assessment of breathlessness in lung/cardiac pathology.
- **Urgent and out-of-hours GP care,** where acute care GPs use PoCUS to rule in/out diverse acute pathologies, for example, deep vein thrombosis (DVT), abscesses, with the intention of accelerating decision processes and minimising unnecessary inpatient admissions.
- **Referral triage**, usually locally commissioned as an enhanced service. Examples include GP-delivered neck lump pathways to improve appropriateness of referral to secondary care head/neck services, and cardiac visualisation in congestive cardiac failure monitoring.
- **Remote/isolated practice**. PoCUS is being used by GPs on the Isles of Scilly to assist decisions on the need for emergency medical transfer to mainland hospital services.
- **Private GP services**, where PoCUS is being included within consultations to enhance diagnosis and direct interventions within the consultation, including minor surgery and joint injections.

### Research

Primary care PoCUS is supported by around 25 years of peer-reviewed studies published in high-impact international medical journals.^2^ Current UK-based studies are limited. Early-stage projects are building on international findings, mapping applications, user profiles, and facilitators and barriers to PoCUS implementation within NHS primary care, in addition to considering novel applications, such as AI-supported diagnosis of DVT.^9,10^ Patient and public involvement activities have demonstrated significant support for targeted GP-delivered ultrasound in the UK, if delivered in a safe and equitable way.^^10^^ Service evaluation data from individual RCGP SIG members suggest potential for reduced radiology and emergency department referrals, time to diagnosis, and low levels of incidental findings/adverse events.

### Policy and governance

Ultrasound is a user-dependent investigation requiring training and continuing medical education to maintain competency, meet local population health needs, and fulfil national regulatory requirements.^2^ The UK General Medical Council requires clinicians to recognise and work within the limits of their competence, practising under supervision appropriate to their role, knowledge, skills and training, and the task undertaken. Medical indemnity providers similarly require demonstration of competency, with premiums equivalent to minor surgery in primary care. International data suggest that PoCUS within primary care has not led to inappropriate or unnecessary care, and may reduce litigation costs.^11^ GPs trained in PoCUS have been shown to have low rates of over- and misdiagnosis when a ‘rule-in’ rather than ‘rule-out’ diagnostic approach is used. Risk of diagnostic error can however increase in clinical scenarios that are not associated with a focused clinical question or if the clinician scans beyond their level of competence. Clear and standardised procedures are required to ensure appropriate handling of incidental findings.

We acknowledge that NHS general practice at present may not be a fertile bed to adopt a root change in diagnostics. As such, it risks falling behind other primary care systems in other countries. International and early UK adopters provide valuable lessons for PoCUS integration, confirming that most scans are conclusive, take ≤5 minutes, and can be integrated within routine GP workflow. Efficient integration is dependent on clear guidelines covering scan indications, training requirements, and reporting/management approaches, including for incidental and unanticipated findings. Governance frameworks for PoCUS in non-radiology settings have been used within UK primary care,^12^ alongside local service contracts with some Integrated Care Boards for specific evidence-based indications. PoCUS training programmes suggest that support from radiology (and other secondary care specialties) at a local and policy level increases GP confidence and accountability in meeting recommended safety, governance, and education frameworks for ultrasound in the UK. Current NHS block-contracting and commissioning structures do not support reallocation of imaging budgets. This raises concerns around PoCUS being designated as unfunded work — a factor hampering innovation across primary care more broadly.

## The future of PoCUS

PoCUS is an emerging clinical tool within general practice globally. It has the potential to enhance traditional clinical assessment by providing rapid, clinician-led diagnostic insight within the consultation. Professional interest and uptake are growing within the UK, shaped by inclusion in undergraduate medical curricula, medical student PoCUS societies, and increasingly compact and affordable devices. Localised training, communities of practice, research, and service evaluations of cost/care impact are supporting a ‘ground up’ implementation in primary care, both in the UK and abroad.

We anticipate a progressive increase in the proportion of UK GPs able to undertake core PoCUS competencies within their routine practice, facilitated by national and locality training and support networks, including the RCGP Ultrasound SIG. The breadth and depth of GP PoCUS applications require consideration to determine the levels of adoption from essential basic competencies to extended roles. Further research to identify where PoCUS is making the greatest impact in clinical outcomes, quality of care, cost savings, and training pathways within primary care would support arguments for inclusion of PoCUS within the MRCGP curriculum. Countries such as Denmark show that acceptance, funding, and trainer capacity considerations for GP PoCUS can resolve on reaching a critical mass of users. Expanding collaborations with radiology and hospital care specialists could support establishment of mutually beneficial ultrasound training rotations/fellowships, referral and governance frameworks. Development of training pathways could promote uptake of specialist PoCUS applications through GP with Extended Role (GPwER) programmes and associated primary care/specialty societies, such as ultrasound-guided minor surgery, sports medicine, women’s health, and dermatology in primary care.

While significant hurdles to widespread adoption exist, we believe that policy shifts towards community-based diagnostics are creating conditions for the successful adoption of PoCUS. Perhaps it’s time to step beyond the stethoscope in UK primary care.
